# Demographic, health, physical activity, and workplace factors are associated with lower healthy working life expectancy and life expectancy at age 50

**DOI:** 10.1038/s41598-024-53095-z

**Published:** 2024-03-11

**Authors:** Marty Lynch, Milica Bucknall, Carol Jagger, Andrew Kingston, Ross Wilkie

**Affiliations:** 1 School of Medicine, Keele University, David Weatherall Building, Newcastle under Lyme, ST5 5BG UK; 2 MRC Versus Arthritis Centre for Musculoskeletal Health and Work, University of Southampton, Southampton, UK; 3 Population Health Sciences Institute, Newcastle University, Newcastle, UK

**Keywords:** Diseases, Risk factors

## Abstract

Although retirement ages are rising in the United Kingdom and other countries, the average number of years people in England can expect to spend both healthy and work from age 50 (Healthy Working Life Expectancy; HWLE) is less than the number of years to the State Pension age. This study aimed to estimate HWLE with the presence and absence of selected health, socio-demographic, physical activity, and workplace factors relevant to stakeholders focusing on improving work participation. Data from 11,540 adults in the English Longitudinal Study of Ageing were analysed using a continuous time 3-state multi-state model. Age-adjusted hazard rate ratios (aHRR) were estimated for transitions between health and work states associated with individual and combinations of health, socio-demographic, and workplace factors. HWLE from age 50 was 3.3 years fewer on average for people with pain interference (6.54 years with 95% confidence interval [6.07, 7.01]) compared to those without (9.79 [9.50, 10.08]). Osteoarthritis and mental health problems were associated with 2.2 and 2.9 fewer healthy working years respectively (HWLE for people without osteoarthritis: 9.50 years [9.22, 9.79]; HWLE with osteoarthritis: 7.29 years [6.20, 8.39]; HWLE without mental health problems: 9.76 years [9.48, 10.05]; HWLE with mental health problems: 6.87 years [1.58, 12.15]). Obesity and physical inactivity were associated with 0.9 and 2.0 fewer healthy working years respectively (HWLE without obesity: 9.31 years [9.01, 9.62]; HWLE with obesity: 8.44 years [8.02, 8.86]; HWLE without physical inactivity: 9.62 years [9.32, 9.91]; HWLE with physical inactivity: 7.67 years [7.23, 8.12]). Workers without autonomy at work or with inadequate support at work were expected to lose 1.8 and 1.7 years respectively in work with good health from age 50 (HWLE for workers with autonomy: 9.50 years [9.20, 9.79]; HWLE for workers lacking autonomy: 7.67 years [7.22, 8.12]; HWLE for workers with support: 9.52 years [9.22, 9.82]; HWLE for workers with inadequate support: 7.86 years [7.22, 8.12]). This study identified demographic, health, physical activity, and workplace factors associated with lower HWLE and life expectancy at age 50. Identifying the extent of the impact on healthy working life highlights these factors as targets and the potential to mitigate against premature work exit is encouraging to policy-makers seeking to extend working life as well as people with musculoskeletal and mental health conditions and their employers. The HWLE gaps suggest that interventions are needed to promote the health, wellbeing and work outcomes of subpopulations with long-term health conditions.

## Introduction

Life expectancy increases in recent decades have prompted policies to defer retirement and extend working lives in many countries around the world ^1^. Work participation has marked benefits for health, affording status and purpose to individuals whilst enabling financial independence, provided it is engaging, safe, secure and offers opportunities for personal progression^2,3^. If not, work can be associated with poorer health, for example through job stressors, the nature of the working role, hazardous exposures and exacerbation of health conditions^4^.

Many countries are increasing pension ages in efforts to extend working lives and defer retirement^5^. Extending working lives implies the need to maintain a healthy workforce. Poor health is a leading cause of departure from the workforce before retirement age in adults aged 50 and over^6–8^. Although the United Kingdom (UK) State Pension age is determined primarily based on life expectancy^9^, changes in life expectancy and changes in health expectancies from birth do not necessarily corresponded to health improvements for adults later in life; expansions of morbidity have been observed in several European countries, while compressions of morbidity have been observed in some others^10^.

Findings from recent UK studies question whether population health is sufficient to indicate that people are able to extend their working lives and work until they are older^11–13^. Using life expectancy projections, the UK State Pension age is legislated ten years in advance such that the duration of State Pension receipt is capped at 32% of adult life on average across the population in order to maintain intergenerational fairness of the ‘pay as you go’ system that funds current retirees using the National Insurance contributions from current workers^14^. Whilst State Pension age in the UK is scheduled to rise to 68 by 2046, the average number of years that people are healthy and in work (Healthy Working Life Expectancy; HWLE) in England is fewer than 10 years from age 50^11^. Furthermore, life expectancy improvements in the UK have slowed since 2011^15,16^. Inequalities in life expectancy and healthy life expectancy at birth, age 50, and age 65 have widened between 2011–2013 and 2018–2020 for people living in the most and least deprived areas of England^17^. Life expectancy has decreased over this period for those living in the most deprived areas of the England (for males and females at birth, age 50, and at age 65)^17^. Healthy life expectancy from birth is similar in the UK overall (in both 2017–19 and 2018–2020) to estimates in 2011–13, reflecting increases and decreases over this time for some UK subpopulations by age group, sex, and constituent country^15^.

HWLE is estimated using both morbidity data and mortality data to quantify the health expectancy (that is, the expected average lifespan in a defined health state) for being healthy and working from age 50^18,19^. HWLE was introduced as a population indicator for monitoring the ongoing sustainability of national social security systems and the quality of later-working-age life through conceptual linkage with markers of successful ageing (health, longevity, and productive engagement through employment)^18^. The potential to extend working lives and be healthy and in work is dependent on a number of health, demographic, lifestyle and workplace factors, and their co-occurring relationships^20–24^. If life expectancy increases but the share of older adults’ lives being spent in good health does not, or there are insufficient suitable work opportunities, it cannot be assumed that adults are automatically able to lengthen their working lives in line with increases to life expectancy at birth. However, inequalities in HWLE and differences associated with factors that may be influenced by policy point to potential to improve health and healthy work participation through targeted interventions and population health initiatives.

The examination of the extent of the influence of specific factors and combinations of factors on remaining healthy and in work is challenging due to computationally intensive methods with high data requirements, and few longitudinal studies with recent data from large sample sizes. Research to meet methodological development needs is ongoing and it has not yet been feasible to develop an overall model of key biopsychosocial drivers of reduced HWLE either in the general population or among specific subgroups of interest to policymakers such as people with musculoskeletal conditions. In this study, we aimed to expand on previously published evidence of differences in HWLE and in life expectancy associated with individual health and sociodemographic factors^11,25^ by quantifying the associations with individual and small combinations factors selected from the wider biopsychosocial model for which data were available and targeted policy initiatives may have leverage.

Models of work disability indicate that there are a range of factors that theoretically reduce work participation; these include demographic, health, lifestyle and workplace factors^4^. In this analysis we selected factors that can be easily assessed and identified by stakeholders focusing on improving work participation, had sufficient data, and could be considered as examples of the broader domains (demographic, health, lifestyle, and workplace) they link to. As well as age and sex, these variables were: obesity; physical inactivity; musculoskeletal conditions, mental health problems, pain interference, inadequate support at work, and lack of autonomy at work. Obesity is a major population health challenge that, due to its prevalence and impact, is associated with lost work participation^26^. Physical inactivity is a risk factor for poor health and function; increasing physical activity is a key target for improving health^27^. Musculoskeletal and mental health problems were included as these are the most common causes of premature work loss and sickness absence (except for recent sickness absence due to covid-19)^4,28,29^. Osteoarthritis was examined as an example of a musculoskeletal disorder. It is the most common musculoskeletal joint condition in adults, the fastest increasing health condition globally, and a leading cause of disability^30,31^. Prevalence and general practice consultation incidence of osteoarthritis sharply increase in age groups over 50^32,33^. A quarter of the UK population aged 50–65 have consulted primary care for osteoarthritis treatment^34^. The impact of osteoarthritis on work is through pain and physical limitation^35^. We therefore included pain interference to capture the main symptom of osteoarthritis and other musculoskeletal conditions. Work factors examined were inadequate support at work and lack of autonomy at work^36–38^.

Through estimation of age-adjusted hazard rate ratios for transitions between health and work states in the healthy working life expectancy model, this study aimed to estimate the extent to which HWLE may be lower for people reporting selected individual-level and time-varying health, physical activity and workplace factors. We report on the extent of the association between these factors and risk of incident ill health/work loss and risk of death among healthy working adults aged 50 and over, and report the corresponding healthy working life expectancy and life expectancy estimates from age 50.

## Methods

**Figure 1:**
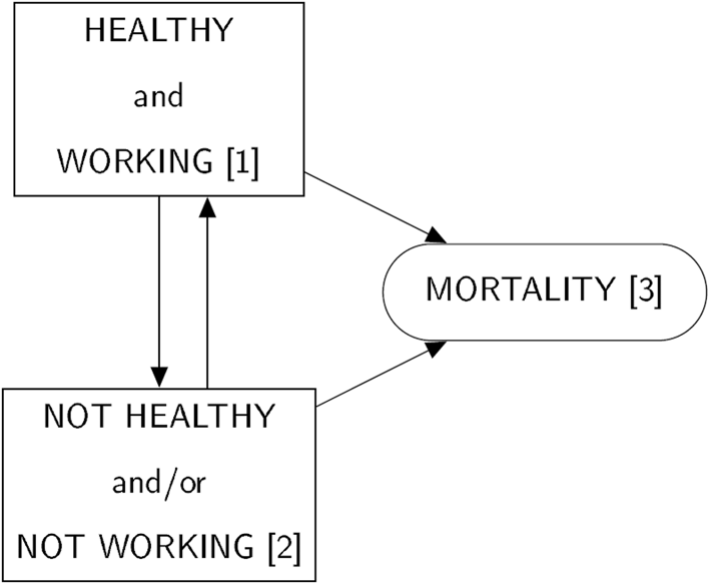
States and permitted transitions (shown with arrow heads) in the 3-state HWLE model.

This study estimated continuous-time multi-state models of hazard rates for transitions between health and work statuses and mortality dependent on year of age (from age 50 to 120) using data collected from adults aged 50 and over in England. Using information about health and work status provided in survey interviews as well as records of death dates for deceased participants, this method models the probabilities with which persons move between states in the multi-state model (Fig. 1) at age 50 and how these probabilities change as age increases from age 50 to end of life (maximum age 120). These models were used to find the corresponding HWLE and life expectancy from age 50 for the general population (using models with age as the only independent variable) and to estimate differences in HWLE and life expectancy associated with the presence or absence of each factor or combination of factors included in separate models including covariates.

### Study design and participants

Data used were from the English Longitudinal Study of Ageing (ELSA) waves 2–6 (2004/5–2012/13)^39^. ELSA has been described in detail elsewhere^40,41^. Briefly, ELSA collects longitudinal survey data from a representative sample of community-dwelling adults aged 50 and over in England. Questions on work factors were asked from ELSA wave 2 (2004/5) onwards. Linked mortality data (year of death) from the National Health Service Central Register are available up to ELSA wave 6 (2012/13); as well as health and work data, survival status and death dates contribute to model estimation for HWLE and life expectancy and so only data up to wave 6 could be used in this study.

This study used data collected from ELSA participants who responded to at least one survey in waves 2–6. Data collected from participants who were aged less than 50 at time of response (for example due to being a younger partner prior to joining the core sample) were excluded as the age scale started at age 50. Participants without multiple observations (either at least two interviews or at least one interview and recorded date of death) were excluded.

### Assessment of health and work statuses

Health and work statuses were self-reported in each survey wave. In line with the UK Equality Act^42^ definition of disability, health was defined by the presence or absence of limiting long-standing illness, obtained from a combination of two survey items: “Do you have any long-standing illness, disability or infirmity?” and, if so, “(Does this/Do these) illness(es) or disability(ies) limit your activities in any way?”. Work was defined as participation in paid work or self-employment within the month preceding the interview.

### Covariates

#### Demographic (age and sex)

Age was measured in years from the midpoint of year of birth (taken as month 6; June) to the month and year of death. Months of death were imputed on the basis of 2010 monthly death rates in England and known vital status at the interview date in the year of death. Sex (male or female) was identified from self-reported ELSA data.

#### Health variables

Osteoarthritis (yes/no) was identified from self-report at present or previous interview of having doctor diagnosed osteoarthritis (or arthritis of an unknown type) ^43,44^. Individuals were classified as having pain interference if they reported often being troubled with pain (“Are you often troubled with pain?” yes/no)^45^.

Mental health problem(s) (yes/no)were identified from a score of 3 or higher on the CESD-8 (Center for Epidemiologic Studies Depression) depression scale and/or reporting having an emotional, nervous or psychiatric problem during the last two years^46–49^. Obesity (yes/no) was defined as BMI ≥ 30 as measured in nurse visits (waves 2, 4 and 6 only)^50^.

#### Lifestyle (physical inactivity)

Individuals were physically inactive (yes/no) if they reported exercising at moderate or vigorous intensity less frequently than twice per week^51,52^.

#### Workplace variables

Inadequate support at work (yes/no) and lack of autonomy at work (yes/no) were identified from responses to self-completion questionnaire statements relating to paid employment in the last month (respondents selected ‘disagree’ or ‘strongly disagree’ to: “I receive adequate support in difficult situations” (support at work); “At work, I feel I have control over what happens in most situations” (autonomy at work)).

### Statistical methods

Continuous-time multi-state models were estimated using the R ‘msm’ package^53^ to estimate baseline hazards (transition intensities between the model states at age 50) and hazard rate ratios (HRRs) for permitted transitions between healthy working states (Fig. 1). In particular, transitions from being healthy and working to being unhealthy and/or not working was of interest, interpreted as the risk to healthy working adults of becoming unhealthy and/or leaving work (Fig. 1). Age was adjusted for in all models to avoid some or all the variables serving to some extent as proxy measures of age. Models were specified conditional on individual or combinations of sociodemographic, health, and workplace factors. Age-adjusted hazard rate ratios (aHRR) were used to examine the association of age and other (binary) covariates with healthy working adults becoming unhealthy and/or leaving work. Associations of age and other covariates were also examined with transitions: unhealthy and/or not working adults becoming both healthy and working; healthy working adults dying; and unhealthy and/or not working adults dying.

Continuous-time multistate models were specified adjusting for age and: sex; OA; mental health; obesity; pain interference; physical activity; autonomy at work; support at work; OA and sex; OA and mental health; OA and autonomy at work; OA and support at work; OA and sex and autonomy at work; OA and sex and support at work. In specifying further models of a health problem with additional covariates, the decision to analyse OA and not mental health was data driven; the confidence interval around the risk of death for healthy working adults with mental health problems was wide, suggesting an infrequently observed transition.

Estimates of aHRRs for the transition of healthy working adults becoming unhealthy and/or leaving work that exceed 1 indicate an association between the modelled variable and increased risk of healthy working adults becoming unhealthy and/or leaving work. However, on their own, estimated aHRRs associated with increased risk to healthy working adults becoming unhealthy and/or leaving work cannot definitively imply an overall reduction in HWLE because the 3-state model contains a route for adults to return to being healthy and working. Therefore, the continuous-time health expectancy approach ‘elect’ (‘estimation of life expectancies using continuous-time multi-state survival models’)^54^ was used to estimate HWLE using hazard results and thereby demonstrate the associations between the modelled variables and overall HWLE. In-built functionality within the ‘msm’ R package to infer time spent in each state is suitable for less complex models, but hazards that do not change with age are unsuitable for life expectancy or health expectancy estimation. ‘Elect’ was developed to provide an approach to estimate health expectancies from continuous time multi-state models that allow hazards to change with age through inclusion of age as a model covariate. Although ‘elect’ is designed for use with time-independent (fixed) covariates, the approach is practicable for demonstrating the direction of association of HWLE with time-varying covariate observations by assuming time-independence in health expectancy estimates. HWLE at age 50 for England overall was estimated by modelling transitions on age without any additional covariates. Life expectancy from age 50 is the sum of HWLE and health expectancies not healthy and/or not working.

### Missing data imputation

Missing data (in health status, work status, or covariate status at each wave) was handled using multiple imputation by predictive mean matching (PMM) across all waves simultaneously and using auxiliary variables identified as predictive of non-response in ELSA technical reports^40,41,55,56^. Full details of missing data handling are provided in supplementary material. Twenty imputed datasets were generated^57,58^; ‘msm’ and ‘elect’ analysis were carried out for each imputed dataset and results were pooled using Rubin’s rules based on source code for functions ‘barnard.rubin’ and ‘pool.scalar’ for pooling univariate parameters in the R ‘mice’ package^59,60^.

Because missing data were imputed 20 times, the imputed value could differ across imputed datasets. Different imputed values for small numbers of individual missing data cases affected convergence success implying an insufficient sample size for estimating more complex models. Cases of failed convergence ranged from one to four (of the 20 imputed datasets) for five of the models specified; these were excluded when pooling model results.

### Sensitivity analyses

To assess whether the 3-state HWLE model was an appropriate simplification of the full 5-state model (with states reflecting all four combinations of the binary health and work states)^11^, HWLE was estimated from the 5-state model including age and no other covariates. We also performed a complete-case sensitivity analysis without imputation of missing data.

### Ethics and inclusion statement

Ethical approval for ELSA is obtained for each survey wave and was obtained from the NRES Committee South Central—Berkshire (11/SC/0374) for wave 6.

### Consent to participate

ELSA respondents gave their informed consent to participate in the study and for mortality data linkage.

This research was carried out in accordance with STROBE guidelines for observational studies^61^.

## Results

The sample size for analysis was 11,540 adults (5251 males and 6289 females), all of whom had multiple observations during ELSA waves 2–6 (either at least two interviews or at least one interview and recorded date of death) while aged 50 years or over (Table 1). At the first survey wave included in the analysis (ELSA wave 2), 27% of respondents were healthy and in work (Tables 1, 2).

**Table 1:** Participant characteristics at the first survey wave included in the analysis with column percentages (ELSA wave 2, N = 8105).

Variable		n (column %)		
		Female	Male	Total
State				
	Healthy and in work	1039 (23)	1131 (31)	2170 (27)
	Healthy and not in work	1743 (39)	1227 (34)	2970 (37)
	Not healthy and in work	239 (5)	204 (6)	443 (5)
	Not healthy and not in work	1452 (32)	1063 (29)	2515 (31)
	Missing health and/or work	3 (< 1)	4 (< 1)	7 (< 1)
	Total	4476	3629	8105
Age at interview				
	50–60 years	1351 (30)	1103 (30)	2454 (30)
	60–70 years	1391 (31)	1197 (33)	2588 (32)
	70–80 years	1100 (25)	928 (26)	2028 (25)
	80–90 years	578 (13)	363 (10)	941 (12)
	90–100 years	56 (1)	38 (1)	94 (1)
	Total	4476	3629	8105

Note: Percentages may not sum to 100% due to rounding.

**Table 2:** Descriptive statistics of health status, working status, and covariates for participants at the first survey wave included in the analysis with row percentages (ELSA wave 2, N = 8105).

Variable		n (row %)		
		Yes	No	Missing
Healthy		5140 (63)	2958 (36)	7 (< 1)
Working		2615 (32)	5490 (68)	0 (0)
Osteoarthritis		1906 (24)	6198 (76)	1 (< 1)
Mental health problem(s)		2015 (25)	5978 (74)	112 (1)
Obese		1935 (24)	4815 (59)	1355 (17)
Pain interference		2253 (28)	5842 (72)	10 (< 1)
Physically inactive		2977 (37)	5124 (63)	4 (< 1)
Lack of autonomy at work (among workers, n = 2615)		435 (17)	1765 (67)	415 (16)
Inadequate support at work (among workers, n = 2615)		534 (20)	1651 (63)	430 (16)

Note: Percentages may not sum to 100% due to rounding.

### Risk of moving from healthy and in work to unhealthy or out of work

**Figure 2:**
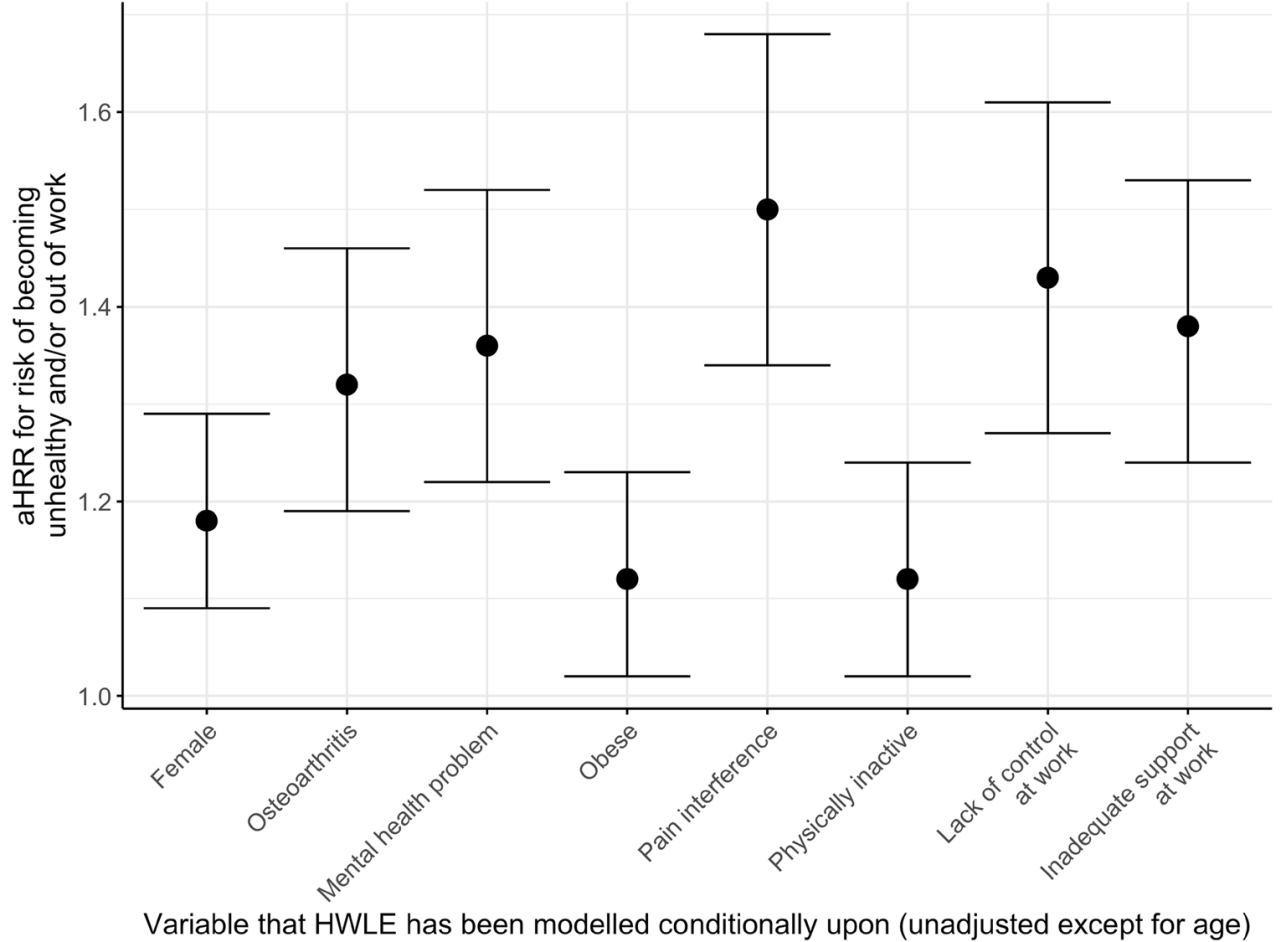
Age-adjusted hazard rate ratios for the risk of incident ill-health/not in work for people aged 50 and over with characteristics: female sex; osteoarthritis; mental health problem; obese; pain interference; physically inactive; don’t have autonomy of work; inadequate support at work. Variables were modelled individually and adjusted for age only.

For every additional year from age 50 onwards, there is a 7% increased risk of becoming unhealthy and/or stopping work (Hazard Rate Ratio (HRR) 1.07; 95% confidence interval 1.06, 1.08) (Table 3). Each of the demographic, health, lifestyle, and workplace factors were associated with higher risk of becoming unhealthy or out of work after adjusting for age (Table 3, Fig. 2). The age adjusted Hazard Rate Ratios (aHRR) for incident ill health/work loss and for return to the healthy working state remained similar for the factors that were analysed together as part of more complex models (Table 3), leading to further reduced time spent healthy and in work on average in populations with multiple challenges to health and/or work participation (Table 3).

**Table 3:** Adjusted hazard rate ratios (adjusted for age and covariates) for transitions: from healthy and working (HW) to unhealthy and/or not working (nHW); from healthy and working to dead; from unhealthy and/or not working to healthy and working; from unhealthy and/or not working to death.

Model covariates	Transition*	Age (95% CI)	Variable 1 (95% CI)	Variable 2 (95% CI)	Variable 3 (95% CI)
(Age only)		Age			
	HW-nHW	1.07 (1.06,1.08)			
	HW-death	1.06 (0.98,1.15)			
	nHW-HW	0.86 (0.85,0.87)			
	nHW-death	1.10 (1.10,1.11)			
Sex		Age	Sex female		
	HW-nHW	1.07 (1.07,1.08)	1.18 (1.09,1.29)		
	HW-death	1.02 (0.93,1.11)	0.12 (0.02,0.90)		
	nHW-HW	0.86 (0.85,0.87)	0.64 (0.56,0.73)		
	nHW-death	1.10 (1.10,1.11)	0.67 (0.61,0.74)		
OA		Age	Has OA		
	HW-nHW	1.07 (1.06,1.08)	1.32 (1.19,1.46)		
	HW-death	1.08 (1,1.17)	0.19 (0.00,21.77)		
	nHW-HW	0.86 (0.85,0.87)	0.66 (0.57,0.77)		
	nHW-death	1.10 (1.10,1.11)	0.89 (0.8,0.98)		
Mental health		Age	Has mental health problem		
	HW-nHW	1.07 (1.06,1.08)	1.36 (1.22,1.52)		
	HW-death	1.06 (0.99,1.14)	0.01 (0, > 1000)		
	nHW-HW	0.85 (0.84,0.86)	0.51 (0.43,0.59)		
	nHW-death	1.10 (1.09,1.11)	1.63 (1.47,1.81)		
Obesity		Age	Obese		
	HW-nHW	1.07 (1.06,1.08)	1.12 (1.02,1.23)		
	HW-death	1.06 (0.97,1.15)	1.59 (0.48,5.27)		
	nHW-HW	0.86 (0.85,0.87)	0.91 (0.79,1.04)		
	nHW-death	1.10 (1.10,1.11)	1.00 (0.89,1.13)		
Pain interference		Age	Has pain interference		
	HW-nHW	1.07 (1.06,1.08)	1.50 (1.34,1.68)		
	HW-death	1.07 (0.98,1.16)	1.60 (0.50,5.13)		
	nHW-HW	0.85 (0.84,0.86)	0.57 (0.49,0.66)		
	nHW-death	1.10 (1.10,1.11)	1.25 (1.13,1.39)		
Physical inactivity		Age	Physically inactive		
	HW-nHW	1.07 (1.06,1.08)	1.12 (1.02,1.24)		
	HW-death	1.07 (0.99,1.15)	0.73 (0.21,2.58)		
	nHW-HW	0.86 (0.85,0.87)	0.55 (0.47,0.64)		
	nHW-death	1.09 (1.08,1.10)	2.27 (2.03,2.53)		
Lack of autonomy at work		Age	No autonomy		
	HW-nHW	1.07 (1.07,1.08)	1.43 (1.27,1.61)		
	HW-death	1.06 (0.98,1.15)	1.75 (0.38,8.02)		
	nHW-HW	0.86 (0.85,0.87)	1.00 (fixed)**		
	nHW-death	1.10 (1.10,1.11)	1.00 (fixed)**		
Inadequate support at work		Age	No support		
	HW-nHW	1.07 (1.07,1.08)	1.38 (1.24,1.53)		
	HW-death	1.06 (0.98,1.15)	1.81 (0.47,6.97)		
	nHW-HW	0.86 (0.85,0.87)	1.00 (fixed)**		
	nHW-death	1.10 (1.10,1.11)	1.00 (fixed)**		
OA + sex		Age	Has OA	Female sex	
	HW-nHW	1.07 (1.06,1.08)	1.28 (1.16,1.42)	1.16 (1.07,1.26)	
	HW-death	1.02 (0.93,1.13)	0.70 (0.1,4.88)	0.14 (0.02,0.97)	
	nHW-HW	0.86 (0.85,0.87)	0.70 (0.60,0.81)	0.67 (0.58,0.76)	
	nHW-death	1.10 (1.10,1.11)	0.93 (0.84,1.03)	0.68 (0.61,0.75)	
OA + mental health		Age	Has OA	Has mental health problem	
	HW-nHW	1.07 (1.06,1.08)	1.30 (1.17,1.44)	1.35 (1.21,1.51)	
	HW-death	1.07 (1.00,1.15)	0.56 (0.10,3.16)	0.01 (0, > 1000)	
	nHW-HW	0.85 (0.84,0.86)	0.71 (0.61,0.83)	0.53 (0.45,0.62)	
	nHW-death	1.10 (1.10,1.11)	0.82 (0.74,0.91)	1.68 (1.51,1.86)	
OA + autonomy at work		Age	Has OA	No autonomy	
	HW-nHW	1.07 (1.06,1.08)	1.32 (1.19,1.47)	1.44 (1.28,1.62)	
	HW-death	1.08 (1.00,1.17)	0.22 (0.00,19.59)	1.59 (0.33,7.70)	
	nHW-HW	0.86 (0.85,0.87)	0.67 (0.57,0.78)	1.00 (fixed)**	
	nHW-death	1.10 (1.10,1.11)	0.89 (0.80,0.98)	1.00 (fixed)**	
OA + support at work		Age	Has OA	No support	
	HW-nHW	1.07 (1.06,1.08)	1.33 (1.2,1.47)	1.39 (1.25,1.54)	
	HW-death	1.08 (1.00,1.17)	0.22 (0.00,18.58)	1.61 (0.42,6.16)	
	nHW-HW	0.86 (0.85,0.87)	0.67 (0.57,0.78)	1.00 (fixed)	
	nHW-death	1.10 (1.10,1.11)	0.89 (0.80,0.98)	1.00 (fixed)	
OA + sex + lack of autonomy at work		Age	Has OA	Sex female	No autonomy
	HW-nHW	1.07 (1.06,1.08)	1.29 (1.16,1.43)	1.16 (1.06,1.26)	1.43 (1.27,1.61)
	HW-death	1.03 (0.94,1.13)	0.77 (0.12,4.80)	0.13 (0.02,0.96)	1.98 (0.40,9.94)
	nHW-HW	0.86 (0.85,0.87)	0.70 (0.60,0.82)	0.67 (0.59,0.76)	1.00 (fixed)**
	nHW-death	1.10 (1.10,1.11)	0.93 (0.84,1.03)	0.68 (0.62,0.75)	1.00 (fixed)**
OA + sex + inadequate support at work		Age	Has OA	Sex female	No support
	HW-nHW	1.07 (1.06,1.08)	1.29 (1.16,1.43)	1.18 (1.08,1.28)	1.40 (1.26,1.56)
	HW-death	1.03 (0.93,1.13)	0.75 (0.11,5.01)	0.14 (0.02,0.98)	1.58 (0.48,5.26)
	nHW-HW	0.86 (0.85,0.87)	0.70 (0.60,0.82)	0.67 (0.59,0.77)	1.00 (fixed)**
	nHW-death	1.10 (1.10,1.11)	0.93 (0.84,1.03)	0.68 (0.62,0.75)	1.00 (fixed)**

Notes:

States: HW Healthy and in work (HWLE) ^1^; nHW Not healthy and/or not in work (including: healthy and not in work; not healthy and in work; not healthy and not in work) ^2^; death ^3^

*Transitions:

“HW-nHW” From healthy and working to unhealthy and/or not working (transition1-2)

“HW-death” From healthy and working to death (transition 1–3)

“nHW-HW” From unhealthy and/or not working to healthy and working (transition 2–1)

“nHW-death” From unhealthy and/or not working to death (transition 2–3)

**aHRRs were fixed at 1 for workplace factors for transitions out of the nHW state (that is, the variable does not affect the risks of transitions out of the state). This is because individuals in the nHW state were not necessarily in work (and workplace factors are not applicable to non-working individuals).

### Risk of mortality

As age increased, the risk of mortality for those who were unhealthy and/or not working increased (HRR 1.10 [1.10, 1.11]). The increased risk of mortality with each additional year of age was (non-significantly) less for those in who were healthy and in work (HRR 1.06 [0.98, 1.15]). With each additional year of age, unhealthy and/or not working adults became less likely to become both healthy and working for pay (HRR 0.86 [0.85, 0.87]). Significantly reduced risks of death were found for women from either alive state (from healthy and in work aHRR 0.12 [0.02, 0.90]; from unhealthy and/or out of work aHRR 0.67 [0.61, 0.74]).

Once in the not healthy and/or not working state, having OA was associated with significantly lower transition rates out of this state (either to join the healthy and working state or the death state). A wide confidence interval surrounded the aHRR for risk of death from the healthy and working state for people with OA, yielding no statistically significant association.

Having a mental health problem was associated with increased risk of death for unhealthy and/or not working people (aHRR 1.63 [1.47, 1.81]).

### HWLE estimates

**Figure 3:**
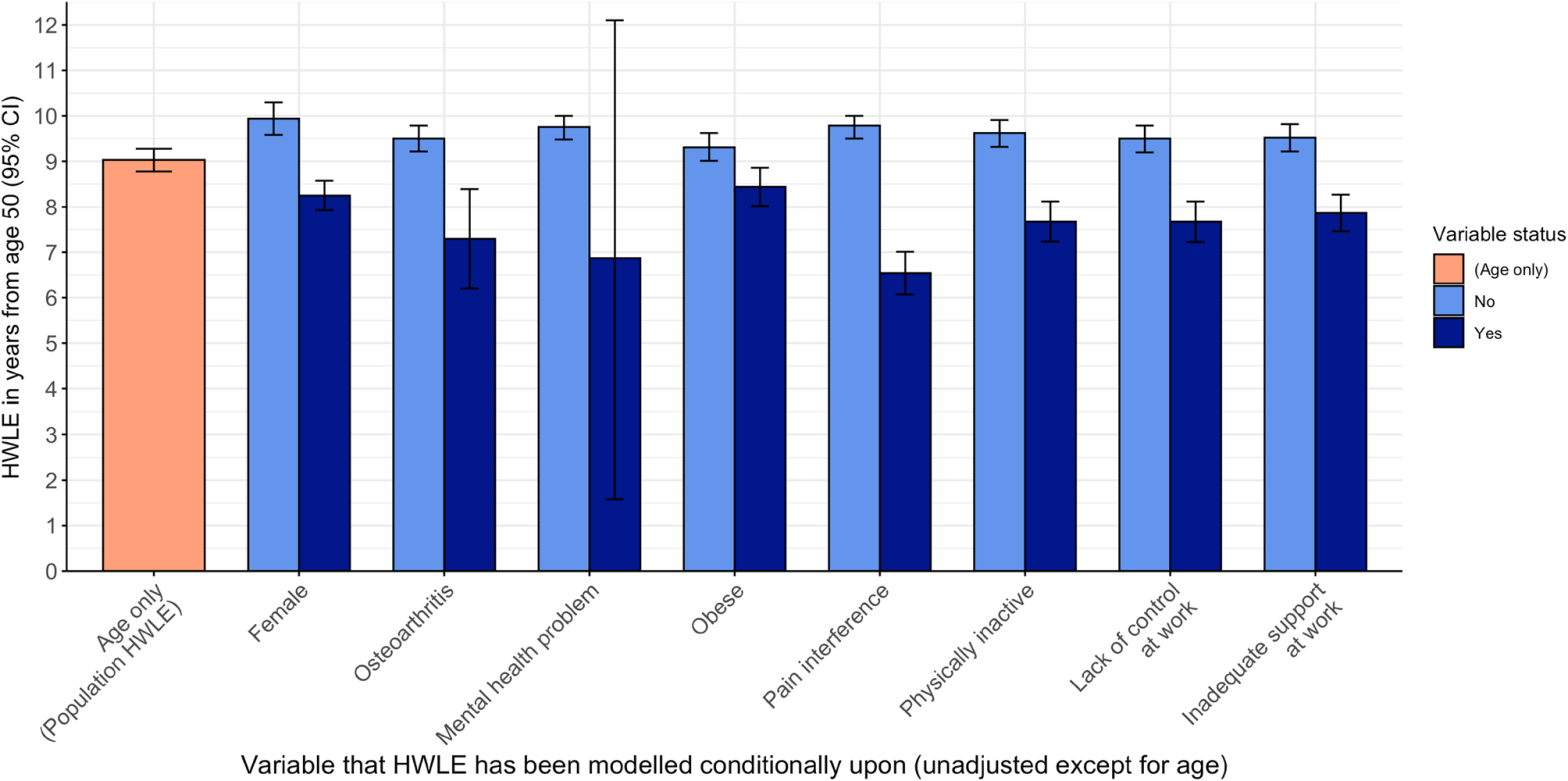
Estimates of HWLE in years from age 50 years with 95% confidence intervals for people with and without characteristics: female sex; osteoarthritis; mental health problem; obese; pain interference; physically inactive; don’t have autonomy at work; inadequate support at work. Variables were modelled individually and adjusted for age only.

HWLE at age 50 for England overall was estimated as 9.03 years (95% CI [8.78, 9.29]). HWLE was significantly higher for men at 9.94 [9.58, 10.31] years and compared to women 8.25 [7.92, 8.58] years (Table 4, Fig. 3).

**Table 4:** Remaining years expected to be spent healthy and in work (HWLE) at age 50 and life expectancy (LE) at age 50 from age-adjusted multi-state models estimated with covariates.

Model	HWLE (95% CI)	Years not healthy and/or not in work (95% CI)	Life expectancy (95% CI)
(Age only)	9.03 (8.78,9.28)	22.59 (22.11,23.07)	31.62 (31.17,32.07)
Sex			
Female	8.25 (7.92,8.58)	25.11 (24.39,25.83)	33.36 (32.64,34.07)
Male	9.94 (9.58,10.31)	19.78 (19.13,20.44)	29.73 (29.06,30.39)
OA			
Has OA	7.29 (6.20,8.39)	25.01 (21.30,28.72)	32.30 (27.64,36.96)
No OA	9.50 (9.22,9.79)	21.80 (21.24,22.35)	31.30 (30.77,31.83)
Mental health			
Has mental health problem	6.87 (1.58,12.15)	22.02 (6.94,37.09)	28.89 (8.56,49.21)
No mental health problem	9.76 (9.48,10.05)	23.24 (22.63,23.86)	33.01 (32.42,33.60)
Obesity			
Obese	8.44 (8.02,8.86)	22.94 (21.98,23.90)	31.38 (30.45,32.31)
Not obese	9.31 (9.01,9.62)	22.42 (21.84,22.99)	31.73 (31.18,32.28)
Pain interference			
Has pain	6.54 (6.07,7.01)	23.33 (22.36,24.30)	29.87 (28.90,30.83)
No pain	9.79 (9.50,10.08)	22.62 (22.04,23.19)	32.41 (31.86,32.96)
Physical inactivity			
Physically inactive	7.67 (7.23,8.12)	20.69 (19.96,21.43)	28.36 (27.66,29.07)
Physically active	9.62 (9.32,9.91)	26.39 (25.46,27.32)	36.01 (35.09,36.92)
Lack of autonomy at work			
No autonomy at work	7.67 (7.22,8.12)	23.54 (22.74,24.34)	31.21 (30.41,32.00)
Has autonomy at work	9.50 (9.20,9.79)	22.26 (21.75,22.77)	31.76 (31.28,32.23)
Inadequate support at work			
No support at work	7.86 (7.46,8.27)	23.37 (22.67,24.08)	31.24 (30.55,31.92)
Has support at work	9.52 (9.22,9.82)	22.25 (21.74,22.76)	31.78 (31.30,32.25)
OA + Sex			
Has OA + Female	6.82 (6.33,7.30)	26.70 (25.76,27.65)	33.52 (32.63,34.40)
Has OA + Male	8.20 (7.48,8.93)	21.88 (20.10,23.66)	30.08 (27.94,32.21)
No OA + Female	8.72 (8.36,9.09)	24.44 (23.64,25.24)	33.16 (32.37,33.96)
No OA + Male	10.30 (9.91,10.69)	19.32 (18.62,20.02)	29.62 (28.92,30.33)
OA + Mental health			
Has OA + Has mental health problem	5.61 (1.08,10.13)	23.90 (7.33,40.48)	29.51 (8.47,50.54)
Has OA + No mental health problem	8.06 (7.52,8.60)	26.10 (24.87,27.34)	34.16 (32.91,35.41)
No OA + Has mental health problem	7.26 (1.32,13.20)	21.01 (5.84,36.17)	28.27 (7.24,49.29)
No OA + No mental health problem	10.17 (9.86,10.48)	22.36 (21.71,23.01)	32.53 (31.90,33.15)
OA + Lack of autonomy at work			
Has OA + No autonomy at work	6.09 (5.02,7.15)	25.97 (21.66,30.29)	32.06 (26.85,37.27)
Has OA + Has autonomy at work	7.71 (6.67,8.76)	24.67 (21.35,27.99)	32.38 (28.19,36.57)
No OA + No autonomyat work	8.08 (7.61,8.55)	22.82 (21.93,23.70)	30.90 (30.01,31.80)
No OA + Has autonomy at work	9.99 (9.66,10.33)	21.44 (20.85,22.04)	31.43 (30.86,32.01)
OA + Inadequate support at work			
Has OA + Inadequate support at work	6.23 (5.15,7.31)	25.86 (21.57,30.14)	32.08 (26.88,37.29)
Has OA + Adequate support at work	7.72 (6.68,8.76)	24.67 (21.36,27.97)	32.39 (28.22,36.55)
No OA + Inadequate support at work	8.27 (7.85,8.70)	22.66 (21.88,23.44)	30.94 (30.16,31.71)
No OA + Adequate support at work	10.03 (9.70,10.37)	21.42 (20.82,22.02)	31.46 (30.88,32.03)
OA + Sex + Lack of autonomy at work			
Has OA + Female + No autonomy at work	5.70 (5.17,6.22)	27.62 (26.50,28.73)	33.31 (32.23,34.40)
Has OA + Female + Has autonomy at work	7.22 (6.71,7.73)	26.37 (25.42,27.32)	33.59 (32.71,34.47)
Has OA + Male + No autonomy at work	6.89 (6.01,7.77)	22.63 (19.93,25.34)	29.52 (26.25,32.79)
Has OA + Male + Has autonomy at work	8.63 (7.90,9.35)	21.57 (19.95,23.19)	30.20 (28.26,32.14)
No OA + Female + No autonomy at work	7.42 (6.93,7.91)	25.46 (24.49,26.44)	32.88 (31.92,33.85)
No OA + Female + Has autonomy at work	9.20 (8.79,9.60)	24.08 (23.25,24.90)	33.27 (32.46,34.09)
No OA + Male + No autonomy at work	8.78 (8.18,9.38)	20.08 (18.82,21.35)	28.86 (27.39,30.34)
No OA + Male + Has autonomy at work	10.79 (10.36,11.22)	19.05 (18.32,19.77)	29.84 (29.10,30.59)
OA + Sex + Inadequate support at work			
Has OA + Female + Inadequate support at work	5.76 (5.26,6.27)	27.57 (26.53,28.61)	33.33 (32.34,34.33)
Has OA + Female + Has support at work	7.21 (6.71,7.72)	26.37 (25.44,27.31)	33.59 (32.72,34.45)
Has OA + Male + Inadequate support at work	7.06 (6.27,7.85)	22.63 (20.34,24.93)	29.69 (26.95,32.43)
Has OA + Male + Has support at work	8.70 (7.96,9.45)	21.50 (19.82,23.18)	30.20 (28.20,32.21)
No OA + Female + Inadequate support at work	7.51 (7.04,7.97)	25.40 (24.47,26.33)	32.91 (32.01,33.81)
No OA + Female + Has support at work	9.20 (8.80,9.61)	24.06 (23.25,24.87)	33.27 (32.47,34.07)
No OA + Male + inadequate support at work	9.01 (8.49,9.53)	20.11 (19.09,21.12)	29.12 (27.99,30.25)
No OA + Male + Has support at work	10.89 (10.45,11.34)	18.95 (18.21,19.69)	29.84 (29.08,30.61)

Each of the demographic, health, lifestyle, and workplace factors were associated with lower HWLE (Table 4, Fig. 3). People with OA were estimated to spend 2.2 years less time in work with good health compared to people without OA on average. Pain interference was associated with 3.3 years less time in work with good health. People with mental health problems were estimated to lose 2.9 years in work with good health compared to people without mental health problems. Obesity was associated with 0.8 years less time in work with good health. Physical inactivity was associated with 2 years less time in work with good health. Workers without autonomy or who had inadequate support at work were estimated to spend 1.8 years or 1.7 years less time in work with good health, respectively, than if they did have autonomy or adequate workplace support.

Further reduced HWLE occurred for people with multiple factors associated with less time in work with good health. Women with OA were estimated to spend 1.4 years less time in work with good health than men with OA, and 3.5 years less time in work with good health than men without OA. On average, people with both OA and mental health problems were estimated to lose 4.6 years in work with good health compared to people with neither OA nor mental health problems. Workers with OA and either no autonomy at work or inadequate support at work were estimated to spend almost 4 years less time in work with good health. This gap for workers was wider between men and women; women with OA and either no autonomy or no support at work were estimated to lose an average of around 5 years in work with good health compared to men without OA in work environments where they have autonomy or support respectively.

### Sensitivity analyses

Full details of the results of sensitivity analyses are given in supplementary material. Life expectancy estimates from age 50 were robust to the use of 3- or 5-state HWLE models.

The 5-state model hazard rate ratio estimates led to HWLE estimated as 8.75 years, slightly lower than the 3-state model point estimate of 9.03 (8.78, 9.29). Confidence intervals could not be obtained for health expectancy estimates from the 5-state model. However, similarity of the point estimate to the confidence interval lower bound from the 3-state model implies no likely significant difference. HWLE estimates from starting in the healthy and working state were consistent between the models: 9.73 (9.48, 9.98) years (3-state model); 9.70 years (5-state model).

HWLE estimates were similar in the main analysis performed on imputed datasets and the complete case sensitivity analysis. Inequalities in HWLE linked to osteoarthritis, mental health, and pain interference were non-statistically significantly wider in estimates from imputed data. HWLE estimates by sex, obesity, physical inactivity, lack of autonomy and work, and inadequate support at work tended to be non-statistically significantly higher in the main analysis on imputed data. Life expectancy estimates were lower in the main analysis on imputed data and were closer to the averaged official life expectancy estimates by sex over the study period from 2004 to 2013^16^.

## Discussion

This longitudinal study of 11,540 adults aged 50 and over in England examined associations between selected demographic, health, lifestyle, and workplace factors with risk of transition between different health and work statuses as well as estimated length of healthy working life associated with the presence and absence of these factors. Healthy workers with OA, pain interference, obesity, physical inactivity, mental health problem(s), inadequate support at work, lack of autonomy at work, and who are women face increased risks of incident ill-health and/or work loss, and these characteristics are associated with less time healthy and in work compared to the national average in England.

Having health problems and unfavourable work conditions together was associated with further reduced HWLE compared to estimates from individual (age-adjusted) models. Although adjustment for realistic levels of confounding was unfeasible (unlike the methodologically similar analysis carried out by Cuthbertson et al.^62^), the similarity of covariate aHRRs in unadjusted and more complex models suggests a compounded contribution of multiple challenging factors to length of healthy working life.

On average, HWLE for people with interfering pain was 3.3 years lower compared to those without interfering pain, and 2.5 years lower than the national average. HWLE was estimated as over 1.5 years lower for women than men; a HWLE gender gap is consistent with earlier research^11^ and may reflect differences in a variety of factors including health factors, levels of sickness absence, common occupations, working conditions, and historical State Pension age^23,29,63^.

HWLE was around 2 years lower for people with OA, which was included as an example of a musculoskeletal condition, and for people with mental health problems compared to the national average, and around 3 years lower compared to people without OA or mental health problems respectively. The risk to healthy workers of becoming unhealthy and/or stopping working was 32% higher for people with OA, and 36% higher for people with mental health problems. Each was more strongly linked to lower HWLE than lifestyle factors obesity and physical inactivity (which were both independently associated with health/work loss and reduced HWLE, especially physical inactivity^26,64,65^), but not as strongly as work factors. Mental health issues present a major reason for sickness absence and are associated with work loss and unemployment^29,66^. The primary mechanism for premature work loss among people with OA may be pain, which is associated with poorer health, quality of life, and productivity at and outside of work^67–71^. Pain interference had the largest association with reduced HWLE among the factors investigated, and the risk to healthy workers of health/work loss was 50% higher for those with interfering pain.

The similarity of estimates for people with OA and with autonomy at work and people without OA and autonomy at work highlight the importance of work factors for staying healthy and in work and suggest that targeting factors in the broader biopsychosocial model can lead to improvements in work outcomes for people with health problems. Having autonomy and support at work were associated; approximately a fifth of workers reported having either support or autonomy at work, but not both support and autonomy or neither.

Sensitivity analyses indicated that aHRR results were robust to the use of a simplified 3-state HWLE model compared to the full 5-state model previously published^11,25^. The higher HWLE estimates resulting from alternative ADL-based health operationalisation despite higher age HRR for transition the from healthy and working to unhealthy and/or not working (transition 1–2) and similar age HRRs for other transitions is likely to reflect different proportions of the population classed as a healthy at age 50. Estimates of HWLE were similar to the main results (analysed using imputed data) in the complete case sensitivity analysis.

Retirement age reforms are widespread across the globe with or without linkage to life expectancy. As health, employment, and policy factors vary across national contexts, the numerical point estimates from this study may not apply in different settings. However, the direction, significance and potential size of the gap in HWLE associated with health, demographic, lifestyle and workplace factors indicate that these can be considered as potential targets for interventions to extend working lives in the UK and elsewhere.

The plateauing of life expectancy for the UK overall is the average of the changes observed in the constituent countries and the local areas within them. At the local authority level, 13.5% of male and female subpopulations experienced life expectancy increases from 2011–13 to 2017–19; other groups saw little change with around a third being non-statistically significantly lower in 2017–19 compared to 2011–13. The most recent estimates of life expectancy, including data from 2020 when mortality rates were impacted by the coronavirus (COVID-19) pandemic, show statistically significantly lower life expectancies in around 5% of local area subpopulations compared to 2011–13^72^. Overall, from 2011 to 2020, life expectancy increased in the South West, South East, London, and in males in Northern Ireland^72^. Most other regions of the UK have seen decreases in more recent years (especially for men in the North East, North West, and Yorkshire and The Humber)^72^. Projected life expectancy in the UK at birth, at age 50, and at age 65 in the year 2030 published by UK’s Office for National Statistics have been down-revised with each new release since 2012^16^. These projections of the average amount of time males and females are expected to live from birth, age 50, and age 65 in 2030 have all decreased by over two years since the same projections were estimated using 2012-based data^16^. Widening inequalities, stalling gains, and slow projected increases in life expectancy point to the need for interventions to improve access to extended healthy working lives to keep up with the rising State Pension age. The existence of inequalities is evidence of drivers of positive and negative outcomes; understanding HWLE differences is a step towards interventions to improve access and reduce inequalities in healthy work.

### Strengths and limitations

This study used longitudinal, nationally representative survey data to quantify the contribution of the factors investigated to population HWLE. This study is among the first to apply the ‘msm’ and ‘elect’ R packages together with multiple imputation to estimate hazard rate ratios and associated health/life expectancies^62^. Use of multiple imputation lowered the risk of bias linked to differential non-response. This work makes a methodological contribution on the number and nature of covariates that may feasibly be analysed together using this approach. Smaller differences between subpopulations were observed in this study than were identified previously using the IMaCh approach^11,25^, which is considered to be methodologically sound but has restrictively high data requirements^73,74^.

Age-adjusted HRR confidence intervals for transitions to death for factors OA and mental health—particularly from the healthy working state—were very wide as individuals may more commonly transition to the unhealthy and/or not working state before the end of their life and not directly to death. This uncertainty around risk of death is unlikely to bias HWLE results as death rates are low in these ages. Other studies have demonstrated an association between OA and increased risk of death, which was not detected in this study, possibly due to improved management of comorbidities among healthy workers with OA^75,76^ and possibly linked to the multistate model specification (designed to investigate HWLE primarily) creating lower sample sizes in study aspects giving information on risks of death.

The use of covariate values reported at the start of the transition may misclassify some transitions in which OA was present as non-OA (thereby lowering HWLE for the non-OA group) as doctor diagnosed OA at one time point implies that OA is likely to have been present to some extent in preceding years^77^, thereby ‘diluting’ the apparent associations between HWLE and OA^25^. There was also some inconsistency in self-report of OA across waves^25^. OA was used as one example of a musculoskeletal condition in this analysis; the relationship between HWLE and other musculoskeletal conditions (including inflammatory arthritis such as rheumatoid arthritis) and its symptoms (e.g. back pain) may differ in extent but we don't expect the direction to differ. The definition of "doctor-diagnosed osteoarthritis" applied in this study may lead to people who don't consult healthcare but have the condition being misclassified; this may underestimate the HWLE gap for osteoarthritis. Similarly the approach to identifying mental health problems (using the a validated approach to identify depressions using the CESD-8 and report of an emotional, nervous or psychiatric problem in the previous two years) may underestimate the estimate of HWLE through responders not reporting such symptoms. Having a physical health problem is associated with increased prevalence of mental health problems^78^ and confounding may widen the HWLE gap observed associated with mental health.

That the method is based on transitions required the exclusion from the study sample of ELSA participants who had only one observation; healthy and working people may be overrepresented in the study sample, leading to overestimation of overall population HWLE. As well as obesity, underweight (approximately 1% of the sample and target population) is associated with increased risk of mortality, and classification as obese or not obese may have reduced the difference in transition intensities associated with obesity observed in this study^79,80^. Misclassification may have also affected physical inactivity classification as there is evidence that people tend to overreport the amount of exercise they do^81^. This implies that exercise, even below recommended levels, may be an important factor associated with longer healthy working lives; however, the extent to which the observed HWLE gap is attributable to physical inactivity itself is unclear as correlation with other lifestyle practices such as diet is likely but could not be adjusted for in this study.

It was not possible to develop models with larger numbers of variables or estimate associations adjusted for confounding. As well as the variables we focussed on, others such as psychological factors are also likely to play a role in driving HWLE. For example, people with an internal locus of control are more likely to be healthier, drive favourable work outcomes, and persist in paid work after a health shock^82–85^. This indicates the need for methodological development and datasets with larger sample sizes to allow combinations of factors to be examined.

Although more recent ELSA data could not be analysed in this study due to mortality data linkage only up until wave 6 (2012–13), findings are relevant as life expectancy and healthy life expectancy remain similar on average to estimates from the start of the last decade^72^.

### Implications for policy and practice

Acknowledging the potential role of single biopsychosocial factors and how they combine to impact on HWLE highlights the scope to reduce inequalities through targeted initiatives, and more work is needed to understand the impact of potential interventions as these will likely vary in effectiveness across individuals and subpopulations and in different contexts. These results, which include multiple variables as well as the interplay of numerous factors in various domains in biopsychosocial models of work, may indicate that an approach to improving HWLE is more likely to be effective with consideration of multiple factors simultaneously rather than a single-variable approach. There is a need to better understand the relationship between HWLE and combinations of factors—especially as some will be more and less amenable to intervention.

Early interventions to improve health-related quality of life and mitigate against work loss among people diagnosed with OA and mental health conditions might include approaches that focus on maintaining function (including self-management approaches), initiatives to identify symptoms early, guidance for employers on ways to improve communication with employees, and individual and group educational activities (e.g. coping strategies) intended to equip individuals with the knowledge and confidence to proactively and effectively manage the condition^71,86,87^. The effectiveness of condition-specific interventions may be limited once health conditions are established and this may be when personal and external factors, included in biopsychosocial work models (e.g. autonomy at work), may be more important for improving HWLE. Autonomy at work could include freedom in carrying out work and informal rituals, and opportunities for training to allow job progression and changing job^23,88^. Supportive workplaces have been linked to improved work outcomes in those experiencing musculoskeletal or mental health problems^71,89^. Evidence-based policy is needed to guide employers to support older workers (especially those with long-term health problems) effectively at work to reduce HWLE inequalities and promote healthy working lives. This could be offered through information resources, free training opportunities, incentives to retain workers, and new legislation and policies.

## Conclusion

This study has identified demographic, health, lifestyle and workplace factors that are associated with lower HWLE and that can be used to support identification of target groups and risk factors for interventions. The potential to mitigate against premature work exit should be encouraging to policy-makers seeking to extend working life as well as people with musculoskeletal and mental health conditions and their employers. However, the HWLE gaps observed suggests that interventions are needed to promote the health, wellbeing and work outcomes of subpopulations with long-term health conditions. As State Pension age is deferred, improving health, lifestyle and workplace conditions will be important for people with long-term health conditions but will also be of benefit to the population in general.

### Supplementary Information

Supplementary Information.

## Data Availability

Data from the English Longitudinal Study of Ageing is freely available to researchers from the UK Data Service: https://beta.ukdataservice.ac.uk/datacatalogue/series/series?id=200011. Cited analysis packages are freely available in R software: https://cran.r-project.org/web/packages/msm/index.html; https://cran.r-project.org/web/packages/elect/index.html; https://cran.r-project.org/web/packages/mice/index.html.
