# Supplementary material for: Demographic, health, physical activity, and workplace factors are associated with lower healthy working life expectancy and life expectancy at age 50
Source: Sci Rep. 2024 Mar 11;14:5936. doi: 10.1038/s41598-024-53095-z (PMC10928117; doi:10.1038/s41598-024-53095-z)
Supplement: Supplementary file 1 — Supplementary Information. [file 41598_2024_53095_MOESM1_ESM.docx]

**Demographic, health, physical activity, and workplace factors are associated with lower healthy working life expectancy and life expectancy at age 50**

Marty Lynch, Milica Bucknall, Carol Jagger, Andrew Kingston, Ross Wilkie

**Supplementary material**

1. Missing data handling
2. Sensitivity analyses results

**Missing data handling**

For some individuals in the study sample, values of covariate(s) or health or work status were missing at some ELSA wave time points. It was necessary to impute missing data in order to analyse all observed transitions. Missing data was handled using multiple imputation by predictive mean matching (PMM), which imputes missing observations by sampling values from similar cases (‘neighbours’) [1,2]. In this approach, missing observations are imputed for each variable in turn using each other variable and this process is repeated to achieve convergence; the final iteration produces an imputed dataset. In order for analyses to reflect the uncertainty due to missing data, twenty imputed datasets were generated [3,4]. The results of analysing all imputed datasets were pooled to produce the final results.

Health status or work status (which were later combined to determine HWLE status) were treated as two variables for multiple imputation. OA missingness was examined after OA had been defined as per the description in the main text of the article (that is, carrying data forward and backwards as applicable based on the assumption that OA could be developed but not lost throughout the study period). The percentage of complete observations of variables to be modelled was 79.48% (supplementary tables 1 and 2). Most cases of missingness affected BMI (6251 missing observations, 14.65%) and work factor measurements (support at work and control at work combined had 2571 missing observations, 6.03%); BMI was not measured in waves 2 and 4, and work factors were measured in self-completion questionnaires with lower response rates than the main interviews. Unmeasured work factors for individuals who were not in work at a given time point were not treated as missing.

Supplementary table 1: Patterns of missingness in variables

| Combinations of variables with missing data | Number of variables with missingness | Count (missing observations) | Percentage of observations missing |
| --- | --- | --- | --- |
| (complete) | 0 | 33904 | 79.48% |
| BMI | 1 | 5498 | 12.89% |
| control at work and support at work | 2 | 1927 | 4.52% |
| BMI, control at work and support at work | 3 | 465 | 1.09% |
| depression (CESD-8) | 1 | 318 | 0.75% |
| depression (CESD-8) and BMI | 2 | 198 | 0.46% |
| support at work | 1 | 80 | 0.19% |
| emotional/nervous/psychiatric problem | 1 | 50 | 0.12% |
| pain interference | 1 | 25 | 0.06% |
| depression (CESD-8), control at work, and support at work | 3 | 24 | 0.06% |
| depression (CESD-8), BMI, control at work, and support at work | 4 | 24 | 0.06% |
| health status | 1 | 20 | 0.05% |
| osteoarthritis | 1 | 18 | 0.04% |
| BMI and support at work | 2 | 15 | 0.04% |
| control at work | 1 | 14 | 0.03% |
| emotional/nervous/psychiatric problem, and BMI | 2 | 13 | 0.03% |
| 33 other combinations (observed <10 times) | varies (range 1-9) | 64 | 0.15% |
| Notes: | | | |
| Missingness is shown for all combinations (observed at least 10 times) of model variables | | | |

The missing at random (MAR) assumption was plausible for the study sample with the inclusion of key variables reported in the ELSA technical reports as associated with response and non-response [5–10]:

- sex
- number of people living in household
- health status
- occupation type
- cohort number (baseline sample or refreshment sample)
- response at previous interview(s)
- white or non-white ethnicity
- whether owns own home
- marital status
- smoking status
- education level
- exercise (physical inactivity)

Variables that were additional to those for modelling were added into the imputation model as auxiliary variables. The three work factors not selected for modelling (pressure at work, security at work, and recognition at work) were also included as auxiliary variables. An urban/rural variable was also identified in ELSA technical reports as associated with response however this is not available in the main ELSA datasets.

Supplementary table 2: Number and percentage of missing observations in variables (missingness in auxiliary variables shown in italics)

| Variable | Count (missing observations) | Percentage of observations missing |
| --- | --- | --- |
| BMI | 6251 | 14.65 |
| *security at work* | 2588 | 6.07 |
| support at work | 2554 | 5.99 |
| *pressure at work* | 2518 | 5.9 |
| control at work | 2475 | 5.8 |
| *recognition at work* | 2461 | 5.77 |
| *occupation category* | 709 | 1.66 |
| depression (CESD-8) | 592 | 1.39 |
| *whether owns own home* | 194 | 0.45 |
| *education category* | 105 | 0.25 |
| emotional/nervous/psychiatric problem | 74 | 0.17 |
| pain interference | 48 | 0.11 |
| OA | 32 | 0.08 |
| health | 31 | 0.07 |
| physical inactivity | 18 | 0.04 |
| work | 10 | 0.02 |
| *smoker* | 9 | 0.02 |
| *marital status* | 6 | 0.01 |
| sex | 0 | 0 |
| *ethnicity white* | 0 | 0 |
| *number of people living in household* | 0 | 0 |

Missing data imputation was performed separately using alternative SAH and ADL-based health indicators in order to carry out the sensitivity analyses of hazard rate and HWLE results to the operationalisation of health.

Implementation in R

Missing data were imputed using a wide dataset with wave-specific variables for measured variable (health, work, OA status etc.) at wave 2, 3, 4, 5 and 6 (for example health, denoted health_2, health_3, health_4, health_5, and health_6). The imputation model included variables to construct the outcome (HWLE status, derived from health and work statuses), variables to be analysed as predictor variables for HWLE transitions, and auxiliary variables identified in ELSA technical reports as predictive of non-response (supplementary table 3). All variables were binary except for BMI category (underweight: BMI less than 18.5, normal weight: BMI 18.5-24.9, overweight: BMI 25.0-29.9, obese: BMI 30 or higher), cohort (four categories), education category (less than secondary education, upper secondary and vocational training, tertiary education, other), occupation category (non-manual occupation, manual occupation, self-employed), smoking status (never smoker, current smoker, ex-smoker), marital status (eight categories), and number of residents in household (numeric ranging from 1-11). For the purpose of imputation, work factors (including those that were auxiliary variables) were assigned value 1 (no control at work, no support at work, etc.) at time points where individuals were not in work. Data provided by a proxy interview were used in imputation and then these observations were removed.

BMI was only measured at every second wave (waves 2, 4 and 6). In order to avoid completely missing variables, BMI category at waves 3 and 5 were carried forward from waves 2 and 4 respectively where possible and remaining missing values were carried backwards from waves 4 and 6 respectively. Because BMI at waves 3 (BMI_3) and 5 (BMI_5) consisted of values primarily from waves 2 (BMI_2) and 4 (BMI_4) respectively, BMI_2 and BMI_4 were not used to predict other variables in the imputation model. The decision to use BMI_3 and BMI_5 for prediction instead of BMI_2 and BMI_4 was taken as this avoided collinearity issues. Cohort number was not used to predict BMI_2 as participants who joined ELSA in refreshment cohorts were not yet in the sample at wave 2.

Missing data imputation was carried out in R using type 1 matching, which finds nearest neighbours based on the distance between the predicted value of the observation and drawn values [2]. Missing values were imputed with an observation randomly selected from the ten nearest neighbours [2]. Twenty iterations of imputations were generated for each of the twenty imputed datasets. Trace plots were visually inspected for convergence, indicated by no clear trends in the later iterations [11]. The twenty imputed datasets were cleaned to correct impossible imputed values for variables with restrictions; if a person (who had an OA status later in the study) was imputed to have OA before a time point where they were imputed not to have OA, the earlier imputed OA value was carried forward. All observations at time points that had no interview (or had a proxy interview) were then removed. Composite variables for HWLE status and mental health were constructed, and BMI category was used to generate a binary variable for obesity.

Supplementary table 3: Variables in the imputation model for ELSA data waves 2-6. There were five variables for each item listed (for observations at waves 2, 3, 4, 5 and 6) except items in bold, which were constant for the study period.

| Outcome | Variables used in models | Auxiliary variables for imputation |
| --- | --- | --- |
| health status* | **sex** | **cohort** |
| work status* | OA | **education category** |
|  | depression*** | **ethnicity white** |
|  | psychiatric problem*** | marital status |
|  | BMI category** | number of residents in household |
|  | pain interference | **occupation category** |
|  | physical inactivity | pressure at work |
|  | control at work | recognition at work |
|  | support at work | security at work |
|  |  | smoking |
|  |  | whether owns home |
|  |  | response at each wave |
| Notes: |  |  |
| Observations at each of the five waves were contained in five variables for each item listed (e.g. health_2, health_3, health_4, health_5, and health_6 give health status at waves 2, 3, 4, 5, and 6 respectively) except for bold items which were not wave-specific | | |
| *These items were used to construct a variable for HWLE status | | |
| **This item was used to construct a variable for obesity | | |
| **These items were used to construct a variable for mental health | | |

**Sensitivity analyses results**

The 5-state model hazard rate ratio estimates led to HWLE estimated as 8.75 years, slightly lower than the 3-state model point estimate of 9.03 (8.78,9.29). Confidence intervals could not be obtained for health expectancy estimates from the 5-state model as the confidence intervals for two transitions were uncertain: from unhealthy and not working to healthy and working; and from healthy and working to unhealthy and not working. However, similarity of the point estimate to the confidence interval lower bound from the 3-state model implies no likely significant difference. Estimates of life expectancy from age 50 were similar. HWLE estimates from starting in the healthy and working state were consistent between the models: 9.73 (9.48,9.98) years (3-state model); 9.70 years (5-state model).

The sample size for the sensitivity analysis without imputation was 9,471 participants (34,086 observations) after excluding observations with missing data in variables (except BMI) and then excluding individuals with fewer than two observations (needed to contribute a transition for analysis). We assumed that obesity status (identified from BMI) was unlikely to differ across neighbouring waves in most cases and therefore continued to carry forward BMI into subsequent waves instead of excluding from the study sample all observations from ELSA waves 3 and 5, at which waves BMI was not measured. This decision was taken to avoid loss of a large portion of our data, which could bias the sample and weaken the statistical power of the analysis. HWLE estimates from the complete case analysis were similar to primary results from the main analysis with multiple imputation, as were ranking of HWLE and life expectancy estimates produced by each model. Life expectancy estimates tended to be non-significantly higher compared to primary results. More models and estimates were affected by very wide confidence intervals compared to the primary results.

Supplementary table 4: Sensitivity analyses of starting-state health expectancies to health definition and number of model states

| Sensitivity analysis | Starting state | HWLE | Years healthy and not working | Years unhealthy and working | Years unhealthy and not working |
| --- | --- | --- | --- | --- | --- |
| 3-state model (Main analysis) | HW | 9.73 (9.48,9.98) | 21.95 (21.48,22.43) |  |  |
|  | nHW | 6.22 (5.87,6.56) | 25.16 (24.63,25.69) |  |  |
|  |  |  |  |  |  |
|  |  |  |  |  |  |
|  |  | HWLE* | Years healthy and not working* | Years unhealthy and working* | Years unhealthy and not working* |
| 5-state model | HW | 9.7 | 11.72 | 1.57 | 8.88 |
|  | H & nW | 5.64 | 14.75 | 1.29 | 9.9 |
|  | nH & W | 7.07 | 11.41 | 3.37 | 9.6 |
|  | nH & nW | 2.72 | 11.79 | 0.95 | 14.31 |
| Notes: | | | | | |
| States:   3-state model: 'HW' Healthy and in work (HWLE); 'nHW' Not healthy and/or not in work (including: healthy and not in  work, not healthy and in work, not healthy and not in work) [2]; death [3]  5-state model: 'HW' Healthy and in work (HWLE); 'H & nW' healthy and not working; 'nH & W' not healthy and working;  'nH & nW' not healthy and not working; death | | | | | |
| Results are given in years | | | | | |
| Confidence intervals are shown in parentheses | | | | | |
| *Confidence intervals could not be estimated from 5-state model hazard rate ratio results | | | | | |

Supplementary table 5: Complete case sensitivity analysis estimates of remaining years expected to be spent healthy and in work (HWLE) at age 50 and life expectancy (LE) at age 50 from age-adjusted multi-state models estimated with covariates

| Model | | HWLE (95% CI) | Years not healthy and/or not in work (95% CI) | Life expectancy (95% CI) |
| --- | --- | --- | --- | --- |
| **(Age only)** | | 8.83 (8.18,9.27) | 23.88 (23.21,24.58) | 32.72 (32.15,33.16) |
| **Sex** | |  |  |  |
|  | Female | 8.09 (6.94,8.58) | 26.44 (23.00,27.28) | 34.52 (29.96,35.15) |
|  | Male | 9.74 (9.08,10.27) | 21.15 (20.28,22.03) | 30.89 (30.11,31.49) |
| **OA** | |  |  |  |
|  | Has OA | 7.38 (6.41,7.96) | 26.43 (23.84,27.57) | 33.81 (30.93,34.70) |
|  | No OA | 9.22 (8.51,9.63) | 22.92 (22.12,23.76) | 32.14 (31.46,32.69) |
| **Mental health** | |  |  |  |
|  | Has mental health problem | 7.06 (0.00,7.64) | 22.98 (1.37,23.97) | 30.03 (1.38,30.75) |
|  | No mental health problem | 9.41 (8.79,9.82) | 24.49 (23.60,25.29) | 33.90 (33.20,34.52) |
| **Obesity** | |  |  |  |
|  | Obese | 8.33 (7.57,8.98) | 24.87 (23.59,25.99) | 33.19 (31.92,34.21) |
|  | Not obese | 9.06 (8.38,9.56) | 23.50 (22.73,24.32) | 32.56 (31.90,33.17) |
| **Pain interference** | |  |  |  |
|  | Has pain | 6.77 (5.94,7.44) | 24.57 (21.74,25.55) | 31.33 (27.83,32.16) |
|  | No pain | 9.40 (8.85,9.83) | 23.97 (23.17,24.71) | 33.37 (32.72,33.93) |
| **Physical inactivity** | |  |  |  |
|  | Physically inactive | 7.57 (0.00,8.13) | 22.09 (1.25,22.97) | 29.66 (1.26,30.33) |
|  | Physically active | 9.30 (8.60,9.75) | 27.23 (26.07,28.35) | 36.53 (35.37,37.48) |
| **Lack of autonomy at work** | |  |  |  |
|  | No autonomy at work | 7.47 (6.80,7.99) | 24.76 (23.56,25.60) | 32.24 (31.04,32.90) |
|  | Has autonomy at work | 9.28 (8.55,9.79) | 23.57 (22.86,24.42) | 32.85 (32.24,33.36) |
| **Inadequate support at work** | |  |  |  |
|  | No support at work | 7.59 (6.98,8.11) | 24.70 (23.66,25.54) | 32.29 (31.18,32.89) |
|  | Has support at work | 9.34 (8.75,9.85) | 23.53 (22.74,24.29) | 32.87 (32.30,33.39) |
| **OA + Sex** | |  |  |  |
|  | Has OA + Female | 6.94 (2.69,7.62) | 28.22 (9.24,29.43) | 35.16 (12.26,36.03) |
|  | Has OA + Male | 8.28 (6.99,8.99) | 23.20 (20.34,24.45) | 31.49 (27.84,32.46) |
|  | No OA + Female | 8.47 (1.29,8.91) | 25.55 (4.32,26.48) | 34.02 (5.60,34.73) |
|  | No OA + Male | 10.02 (9.38,10.52) | 20.59 (19.66,21.38) | 30.61 (29.65,31.29) |
| **OA + Mental health** | |  |  |  |
|  | Has OA + Has mental health problem | 6.00 (0.00,6.72) | 25.10 (1.77,26.43) | 31.09 (1.77,32.19) |
|  | Has OA + No mental health problem | 8.00 (7.18,8.59) | 27.47 (25.54,28.66) | 35.48 (33.55,36.49) |
|  | No OA + Has mental health problem | 7.40 (0.00,7.96) | 21.75 (1.21,22.75) | 29.14 (1.23,30.06) |
|  | No OA + No mental health problem | 9.73 (9.17,10.24) | 23.48 (22.64,24.35) | 33.22 (32.40,33.91) |
| **OA + Lack of autonomy at work** | |  |  |  |
|  | Has OA + No autonomy at work | 6.17 (5.11,6.80) | 27.34 (23.18,28.30) | 33.51 (28.88,34.32) |
|  | Has OA + Has autonomy at work | 7.80 (6.82,8.43) | 26.07 (23.53,27.12) | 33.87 (31.23,34.70) |
|  | No OA + No autonomy at work | 7.81 (7.08,8.34) | 23.85 (22.40,24.71) | 31.66 (30.15,32.33) |
|  | No OA + Has autonomy at work | 9.68 (8.99,10.14) | 22.60 (21.81,23.36) | 32.28 (31.59,32.89) |
| **OA + Inadequate support at work** | |  |  |  |
|  | Has OA + Inadequate support at work | 6.22 (5.17,6.88) | 27.31 (22.85,28.63) | 33.53 (28.22,34.43) |
|  | Has OA + Adequate support at work | 7.83 (6.84,8.58) | 26.05 (23.90,27.17) | 33.88 (31.56,34.69) |
|  | No OA + Inadequate support at work | 7.92 (7.28,8.46) | 23.81 (22.59,24.60) | 31.73 (30.64,32.33) |
|  | No OA + Adequate support at work | 9.76 (9.09,10.24) | 22.54 (21.71,23.39) | 32.31 (31.62,32.86) |
| **OA + Sex + Lack of autonomy at work** | |  |  |  |
|  | Has OA + Female + No autonomy at work | 5.84 (0.00,6.47) | 29.19 (1.43,30.27) | 35.03 (1.43,35.95) |
|  | Has OA + Female + Has autonomy at work | 7.35 (0.00,7.97) | 27.89 (1.44,29.01) | 35.24 (1.44,36.15) |
|  | Has OA + Male + No autonomy at work | 6.84 (5.17,7.60) | 23.32 (16.00,25.10) | 30.16 (21.47,31.97) |
|  | Has OA + Male + Has autonomy at work | 8.70 (7.70,9.54) | 22.94 (20.94,24.16) | 31.64 (29.24,32.75) |
|  | No OA + Female + No autonomy at work | 7.22 (0.00,7.69) | 26.59 (0.97,27.46) | 33.81 (0.97,34.52) |
|  | No OA + Female + Has autonomy at work | 8.90 (0.00,9.41) | 25.19 (0.92,26.09) | 34.09 (0.92,34.85) |
|  | No OA + Male + No autonomy at work | 8.47 (7.59,9.12) | 21.10 (18.82,22.27) | 29.57 (26.70,30.53) |
|  | No OA + Male + Has autonomy at work | 10.49 (9.74,11.03) | 20.38 (19.38,21.34) | 30.87 (29.99,31.51) |
| **OA + Sex + Inadequate support at work** | |  |  |  |
|  | Has OA + Female + Inadequate support at work | 5.79 (0.00,6.36) | 29.23 (1.51,30.29) | 35.02 (1.51,35.80) |
|  | Has OA + Female + Has support at work | 7.37 (0.00,7.93) | 27.88 (1.39,28.97) | 35.24 (1.39,36.16) |
|  | Has OA + Male + Inadequate support at work | 6.96 (5.59,7.66) | 23.66 (18.57,25.37) | 30.62 (24.32,32.12) |
|  | Has OA + Male + Has support at work | 8.83 (7.88,9.53) | 22.82 (20.79,24.16) | 31.65 (29.16,32.69) |
|  | No OA + Female + Inadequate support at work | 7.19 (0.00,7.66) | 26.62 (1.15,27.48) | 33.81 (1.15,34.55) |
|  | No OA + Female + Has support at work | 8.96 (0.00,9.51) | 25.15 (0.97,26.00) | 34.10 (0.97,34.85) |
|  | No OA + Male + inadequate support at work | 8.61 (7.83,9.24) | 21.33 (19.81,22.31) | 29.94 (28.37,30.74) |
|  | No OA + Male + Has support at work | 10.66 (9.91,11.20) | 20.25 (19.31,21.06) | 30.91 (29.87,31.58) |

**References**

1. De Silva, A. P., Moreno-Betancur, M., De Livera, A. M., Lee, K. J. & Simpson, J. A. Multiple imputation methods for handling missing values in a longitudinal categorical variable with restrictions on transitions over time: a simulation study. *BMC Med. Res. Methodol.* **19**, 14 (2019).

2. Morris, T. P., White, I. R. & Royston, P. Tuning multiple imputation by predictive mean matching and local residual draws. *BMC Med. Res. Methodol.* **14**, 75 (2014).

3. White, I. R., Royston, P. & Wood, A. M. Multiple imputation using chained equations: Issues and guidance for practice. *Stat. Med.* **30**, 377–399 (2011).

4. Graham, J. W., Olchowski, A. E. & Gilreath, T. D. How many imputations are really needed? Some practical clarifications of multiple imputation theory. *Prev. Sci.* (2007).

5. Banks, J., Nazroo, J. & Steptoe, A. *The dynamics of ageing: Evidence from the English Longitudinal Study of ageing 2002-12 (Wave 6)*. http://www.cemmap.ac.uk/publications/7411 (2014).

6. Banks, J., Nazroo, J. & Steptoe, A. *The dynamics of ageing: evidence from the English longitudinal study of ageing 2002-10 (wave 5)*. (2012).

7. Banks, J. *et al.* *Financial circumstances, health and well-being of the older population in England*. http://www.academia.edu/download/8403620/elsa_w4-1.pdf (2010).

8. Scholes, S., Taylor, R., Cheshire, H., Cox, K. & Lessof, C. *Technical report (ELSA wave 2): retirement, health and relationships of the older population in England*. (2008).

9. Taylor, R. *et al.* *Health, wealth and lifestyles of the older population in England: The 2002 English Longitudinal Study of Ageing Technical Report*. https://www.ifs.org.uk/elsa/report03/w1_tech.pdf (2007).

10. Scholes, S. *et al.* *Living in the 21st century: older people in England*. (2006).

11. van Buuren, S. & Groothuis-Oudshoorn, K. {mice}: Multivariate Imputation by Chained Equations in R. *J. Stat. Softw.* **45**, 1–67 (2011).
